# BUILDing SCHOLARS: enhancing diversity among U.S. biomedical researchers in the Southwest

**DOI:** 10.1186/s12919-017-0095-4

**Published:** 2017-12-04

**Authors:** Timothy W. Collins, Stephen B. Aley, Thomas Boland, Guadalupe Corral, Marc B. Cox, Lourdes E. Echegoyen, Sara E. Grineski, Osvaldo F. Morera, Homer Nazeran

**Affiliations:** 1 Department of Sociology & Anthropology, University of Texas at El Paso, El Paso, TX 79968 USA; 2 Department of Biological Sciences, University of Texas at El Paso, El Paso, TX 79968 USA; 3 Department of Metallurgical & Materials Engineering, University of Texas at El Paso, El Paso, TX 79968 USA; 4 Research Evaluation & Assessment Services, University of Texas at El Paso, El Paso, TX 79968 USA; 5 Campus Office of Undergraduate Research Initiatives and Department of Chemistry, University of Texas at El Paso, El Paso, TX 79968 USA; 6 Department of Psychology, University of Texas at El Paso, El Paso, TX 79968 USA; 7 Department of Electrical & Computer Engineering, University of Texas at El Paso, El Paso, TX 79968 USA

## Abstract

**Background and purpose:**

With funding from the National Institutes of Health, BUILDing SCHOLARS was established at The University of Texas at El Paso with the goal of implementing, evaluating and sustaining a suite of institutional, faculty and student development interventions in order to train the next generation of biomedical researchers from the U.S. Southwest region, where the need is dire among underserved communities. The focus is on supporting the infrastructure necessary to train and mentor students so they persist on pathways across a range of biomedical research fields. The purpose of this article is to highlight the design and implementation of BUILDing SCHOLARS’ key interventions, which offer a systemic student training model for the U.S. Southwest. In-depth reporting of evaluation results is reserved for other technical publications.

**Program and key highlights:**

BUILDing SCHOLARS uses a comprehensive regional approach to undergraduate training through a multi-institution consortium that includes 12 research partners and various pipeline partners across Texas, New Mexico, and Arizona. Through faculty collaborations and undergraduate research training, the program integrates social and behavioral sciences and biomedical engineering while emphasizing seven transdisciplinary nodes of biomedical research excellence that are common across partner institutions: addiction, cancer, degenerative and chronic diseases, environmental health, health disparities, infectious diseases, and translational biomedicine. Key interventions aim to: (1) improve institutional capacities by expanding undergraduate research training infrastructures; (2) develop an intra- and cross-institutional mentoring-driven “community of practice” to support undergraduate student researchers; (3) broaden the pool of student participants, improve retention, and increase matriculation into competitive graduate programs; and (4) support faculty and postdoctoral personnel by training them in research pedagogy and mentoring techniques and providing them with resources for increasing their research productivity. Student training activities focus on early interventions to maximize retention and on enabling students to overcome common barriers by addressing their educational endowments, science socialization, network development, family expectations, and material resources. Over the long term, BUILDing SCHOLARS will help increase the diversity of the biomedical research workforce in the U.S. by meeting the needs of students from the Southwest region and by serving as a model for other institutions.

## The southwest context

The growth of underrepresented minority (URM) populations in the U.S. has not been mirrored by an equitable increase in the number of successful URM biomedical scientists [1]. The nation must address issues of underrepresentation in order to improve the quality of training environments, broaden research priorities, improve the diversity of participants in clinical research protocols, produce adequate numbers of qualified scientists, and improve abilities to address health disparities [2]. BUILDing SCHOLARS seeks to redress the underrepresentation of people from disadvantaged backgrounds in the biomedical sciences by focusing initiatives on the U.S. Southwest region, specifically with institutional partners in the states of Texas, New Mexico and Arizona, which are home to dense concentrations of Hispanic and Native American students. This regional approach provides a hub for transformation with high impact on the targeted population. Although we embrace an all-inclusive approach, the geographic distribution of various underrepresented groups in the U.S. make it difficult to effectively target individuals from all backgrounds. BUILDing SCHOLARS therefore primarily targets Hispanic/Latino and Native American students, but also African Americans, who, in the aforementioned states, are most densely concentrated in east Texas. We refer to our target population as “Southwest underrepresented groups” (SURGes). Table 1 shows the racial/ethnic composition of the regional population. Note the high concentration of Hispanic and Native Americans in the region in comparison to the U.S. figures, which emphasizes the need for a regional focus. In addition, BUILDing SCHOLARS conceives of *biomedical research* as broadly referring to health-related scholarship from natural, behavioral, clinical and social science and engineering fields.

**Table 1:** Demographics of the targeted tri-state region (Source: U.S. Census Bureau, 2015 Population Estimates)

	U.S. Population	% U.S. Population	AZ, NM, TX Population	% AZ, NM, TX Population
Total	321,418,820	–	36,382,288	–
Native American (non-Hispanic)	2,369,834	0.73	545,393	1.50
Black (non-Hispanic)	39,925,949	12.42	3,556,295	9.77
Hispanic	56,592,793	17.61	13,769,769	37.85

The University of Texas at El Paso (UTEP), located on the U.S.-Mexican border, is the primary institution in the BUILDing SCHOLARS network of partners. UTEP’s overall student population as of Fall 2015 is 23,397. Over 87% of UTEP’s 20,220 undergraduate students come from El Paso County, and over 50% of El Paso County’s college-bound high school graduates enroll at UTEP. The ethnic composition of UTEP’s student body mirrors the community it serves, with Hispanics, mostly Mexican-Americans, accounting for nearly 83% of the total undergraduate enrollment. Less than 7% of undergraduate students are White (non-Hispanic), less than 3% are Black (non-Hispanic), and just over 1% are of any other race; international students (more than 78% of whom are Mexican) constitute the remaining 6% of the undergraduate student body. According to institutional records, 57% of enrolled undergraduates at UTEP from 2009 to 2013 were from the first generation in their families to attend college.

Social, cultural and economic challenges limit many UTEP students from moving away from the family home, engaging in research experiences and pursuing advanced degrees. According to 2008–2012 data from the National Survey of Student Engagement administered to UTEP seniors, 56% worked off campus due to financial necessity, 70% took care of dependents living with them, and 33% were enrolled at UTEP less than full-time. According to institutional records, most UTEP students are of low socioeconomic status, with 67% of enrolled undergraduates from 2009 to 2013 receiving federal Pell grants for low-income students. Campus survey data from students participating in undergraduate research training programs from 2009 to 2013 reveal that those who worried about financing their education or worked off campus were less likely to pursue graduate degrees than other students.

Despite the challenges many students face, UTEP is nationally recognized for student engagement and its contribution to diversity in higher education. In Kuh et al.’s book [3], *Student Success in College: Creating Conditions That Matter*, UTEP was presented as a high-impact university in terms of student engagement, since it exhibited both higher-than-predicted graduation rates and higher-than-predicted scores on the National Survey of Student Engagement (NSSE). Additionally, UTEP has a strong record of engaging undergraduate students in research and preparing them for successful science careers. An array of externally and internally funded undergraduate research programs at UTEP have helped many students from underrepresented backgrounds overcome obstacles, including (but not limited to) low incomes and low levels of familial educational attainment. Unpublished data from ten undergraduate research training programs at UTEP reveal that, between 1985 and 2013, 924 undergraduate students were trained through those programs; 80% of those students graduated (or were enrolled and still pursuing an undergraduate degree). Moreover, of the 519 students trained through those programs who had completed their undergraduate degrees, 88% were either pursuing a graduate degree (23%) or had already earned one (65%).

UTEP is also one of the nation’s leading contributors to diversity in higher education, specifically through the provision of educational opportunities for the rapidly growing albeit socio-economically disadvantaged U.S. Hispanic/Latino population. UTEP ranks second among all universities in the continental U.S. in conferring bachelor’s degrees to Hispanic students [4]. In terms of graduate education, UTEP ranks as the top doctoral research university in the U.S. with a Mexican-American majority student population, and is ranked fourth in awarding Master’s degrees to Hispanic students [4]. UTEP has also had a diversifying impact on the nation’s doctoral workforce. National Science Foundation surveys of earned doctorates (2010–2014) rank UTEP 15th as the baccalaureate institution of origin among Hispanic science and engineering doctoral recipients in the U.S. [5]. *Washington Monthly* magazine’s 2015 rankings place UTEP as the tenth best U.S. university overall, which is largely attributable to UTEP’s number one ranking in the “social mobility” category (i.e. providing opportunity for low socioeconomic status SURGes). Thus, prior to BUILDing SCHOLARS, UTEP had long demonstrated a commitment to engaging undergraduate students—most of whom are from SURGes—in training for successful scientific careers.

## The BUILDing SCHOLARS program

### A partnership network

**Fig. 1:**
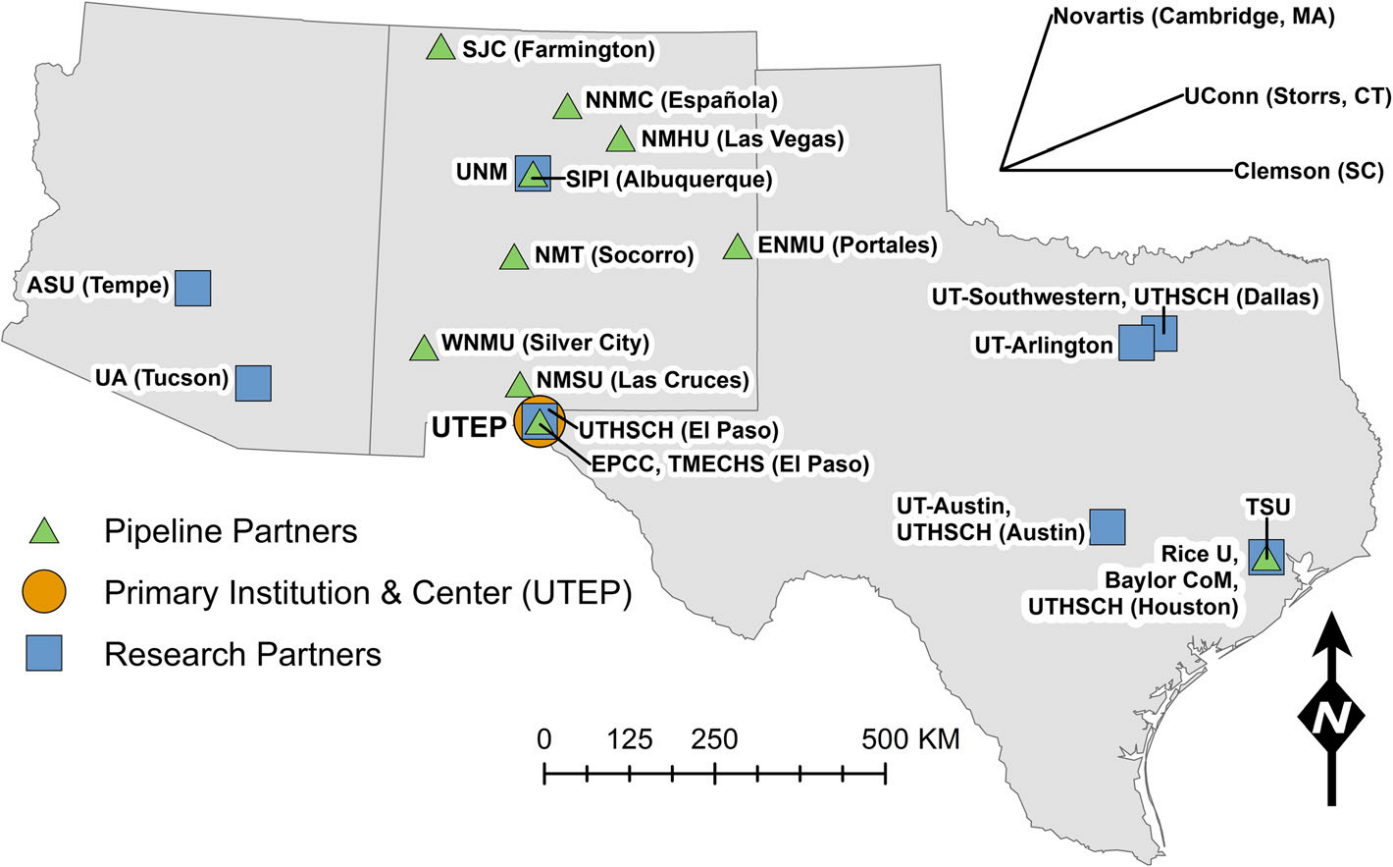
Location of the partner institutions within the regional network

**Fig. 2:**
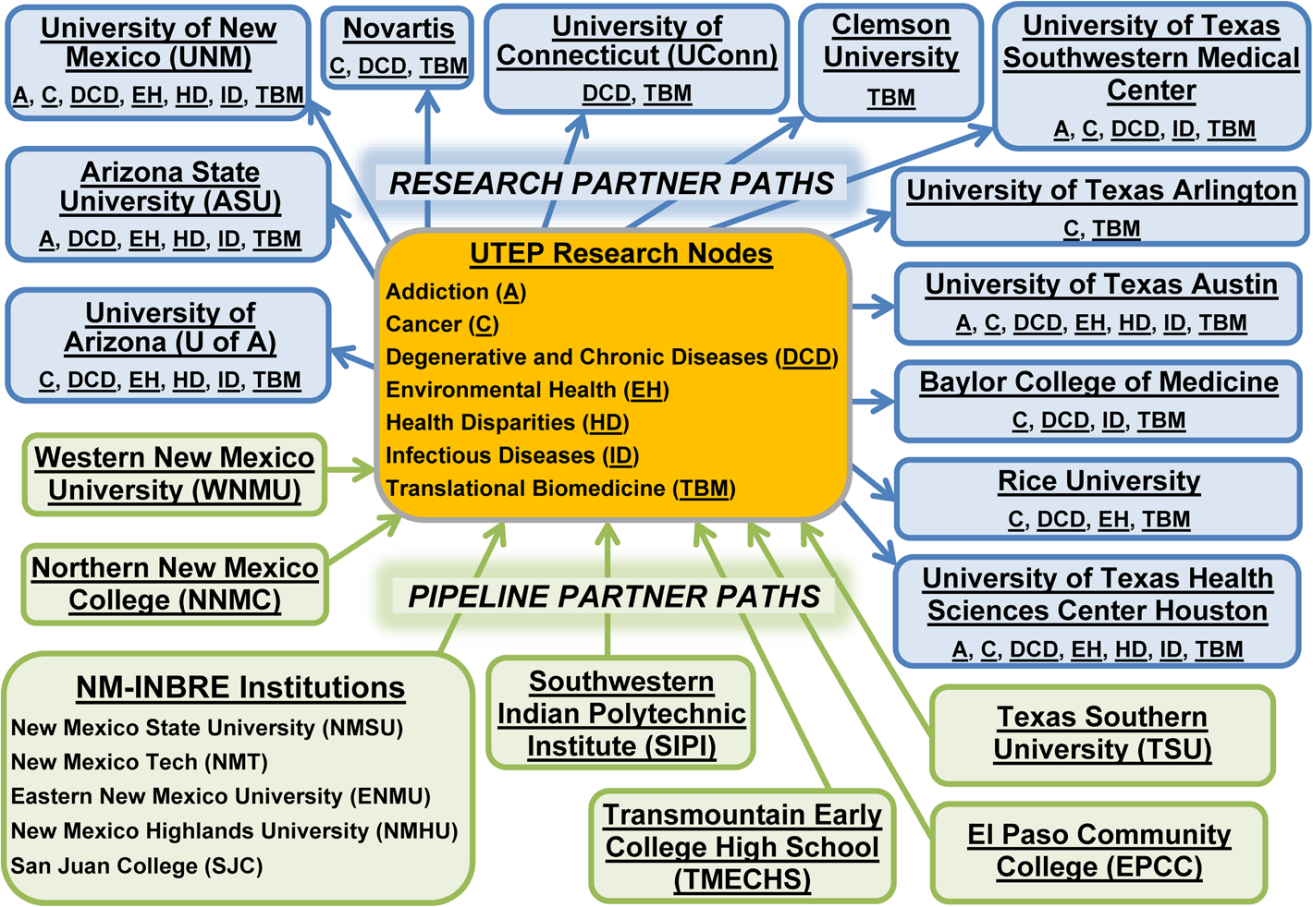
Biomedical research nodes and training paths within the regional network

BUILDing SCHOLARS offers opportunities for student and faculty development across an institutional partnership committed to increasing biomedical research training capacity on a regional scale. Figs. 1 and 2 show the location and roles of the partner institutions within the regional network. The partnership includes UTEP as the primary institution in a network of 12 research, one industrial research, and 11 pipeline partner institutions. Four pipeline partners are two-year and seven are four-year institutions (see Table 2). UTEP has links to five of the pipeline partner institutions through the NIH-funded New Mexico IDeA (Institutional Development Award program) Networks of Biomedical Research Excellence (NM-INBRE) via New Mexico State University. Research Partners were selected based on four criteria: (a) geographic location in the U.S. Southwest (see Fig. 1); (b) large populations of SURGes; (c) excellence in one or more biomedical research nodes corresponding with those at UTEP (see Fig. 2); and (d) a pre-existing relationship with a UTEP BUILDing SCHOLARS program director and/or a track record of recruiting UTEP students. Because not all biomedical research specializations are offered within the region, three extra-regional partners (Clemson University, University of Connecticut, and Novartis Pharmaceuticals) were strategically selected to expand student training opportunities via top-flight academic and private-sector research in translational biomedicine. Our regional, multi-institutional partnership approach nurtures a broader sense of community and promotes the development of emerging biomedical researchers from SURGes by establishing opportunities for faculty, post-doctoral fellows and students through collaborations with world-class research institutions.

**Table 2:** Institutions and roles in the BUILDing SCHOLARS partnership

State	Type of Partner	Institution
AZ	Research	Arizona State University
AZ	Research	University of Arizona
NM	Pipeline	Eastern New Mexico University (through NM-INBRE)
NM	Pipeline	New Mexico Highlands University (through NM-INBRE)
NM	Pipeline	New Mexico Institute of Mining & Technology (through NM-INBRE)
NM	Pipeline	New Mexico State University (through NM-INBRE)
NM	Pipeline	Northern New Mexico College
NM	Pipeline	San Juan College (through NM-INBRE)
NM	Pipeline	Southwestern Indian Polytechnic Institute
NM	Research	University of New Mexico Health Sciences Center
NM	Research	University of New Mexico Main Campus
NM	Pipeline	Western New Mexico University
TX	Research	Baylor College of Medicine
TX	Pipeline	El Paso Community College
TX	Research	Rice University
TX	Pipeline	Texas Southern University
TX	Pipeline	Transmountain Early College High School
TX	Research	UT Arlington
TX	Research	UT Austin
TX	Primary	UT El Paso
TX	Research	UT Health Sciences Center - Houston
TX	Research	UT Southwestern
CT	Research	University of Connecticut
SC	Research	Clemson University
MA	Industrial Research	Novartis

The partnership network offers (a) summer research opportunities for UTEP and pipeline partner undergraduate students, (b) summer sabbaticals and supermentor programs for UTEP and pipeline partner faculty members, and (c) seed funding for faculty across the network to engage in collaborative research and/or research education projects that involve undergraduate students. The 10-week summer research program (SRP) focuses on leveraging network resources to provide research opportunities for students from SURGes. UTEP BUILD scholarship recipients (our trainees) and students from pipeline partner institutions conduct research projects under the mentorship of faculty members across the network. Through corresponding sub-awards, in each grant year, research partners are able to provide 32–84 additional guaranteed world-class summer research experiences to UTEP trainees and pipeline partner students. Note that the numbers of students destined for research partners increase from one summer to the next as the number of trainees increases. In the absence of such guaranteed positions, students would have to compete with applicants from across the nation for positions at those institutions funded by other agencies/programs. Similarly, although UTEP has multiple externally and internally funded summer research programs, BUILDing SCHOLARS provides 35–41 additional summer positions at UTEP each grant year. Approximately half of those UTEP summer positions are reserved for pipeline partner students. In 2016, 82 students were placed in new BUILD-funded summer research experiences; of those, 33 students participated at UTEP (15 of which came from pipeline partners and 18 of which were UTEP BUILD trainees), 34 UTEP BUILD trainees participated at research partners, and 15 students participated at El Paso Community College (EPCC). In 2017, we expect to place 120 students in BUILD-funded summer research experiences.

EPCC is an important pipeline partner and the institution of origin for the vast majority of UTEP transfer students. BUILDing SCHOLARS is providing a sub-award so that EPCC Principal Investigators can offer their own research-driven courses (see the “Faculty Development” and “Preparing Students for Biomedical Research Careers” sections below for further details) and summer research experiences for their affiliated early college high school students. As of spring 2017, 30 students have participated in EPCC BUILD summer research experiences, and 157 students have participated in EPCC BUILD research-driven courses.

The BUILDing SCHOLARS network is transdisciplinary by design. As part of the project planning process, UTEP conducted a capacities and needs assessment, which enabled identification of nodes (i.e., transdisciplinary research areas) of biomedical research expertise among faculty at UTEP that cut across the socio-behavioral, applied/clinical and natural sciences and engineering. The nodes are shown in Fig. 2. Research partners were chosen because of their demonstrated excellence in one or more of those research areas. The research training paths are used to coherently link students interested in those research areas from UTEP and pipeline partners with mentors across the network. The research nodes and training paths also link faculty across the network via summer sabbaticals, the supermentor program, seed funding and other capacity building interactions, in which resources are shared and collaborations fostered. The arrows in Fig. 2 that link institutions depict the general direction of student flows along research training paths between partners.

As a guiding philosophy that orients activities within the partnership, BUILDing SCHOLARs seeks to leverage resources across institutions in order to synergistically expand research training opportunities for SURGes. For example, BUILDing SCHOLARS reserves scholarship and research opportunities specifically for qualified students who complete their two-year degree at pipeline partner institutions and are interested in completing a Bachelor’s degree at UTEP. As a second example, we are striving to create opportunities for BUILDing SCHOLARS graduates to enroll in funded doctoral programs at research partner institutions. The summer experiences with our research partners are not only exposing students to cutting-edge research, but also opening doors for them to pursue doctoral training. The majority of our research partner institutions have multiple NIH-funded T32 training grants; at this writing, 49 T32 training awards were active in the network. In addition to those training programs, all of our research partner institutions have large numbers of NIH R01-funded faculty members with the potential to support our graduates. Across the partnership network, we are also implementing new research-driven curricula as well as faculty development and institutional capacity building activities, all of which are serving to establish and integrate the research nodes and training paths across the institutions involved. The partnership network is effectively engaging and inspiring students from SURGes to pursue excellent training opportunities across a broad range of biomedical science fields, which will contribute to the diversification of the NIH-funded workforce over the long term.

## Institutional development

The institutional development aims of BUILDing SCHOLARS are to: (1) catalyze infrastructure changes needed to implement a large capacity undergraduate research training program; and (2) establish institutional mechanisms to sustain successful program elements beyond the period of NIH support.

### Infrastructure transformation for student learning

**Fig. 3:**
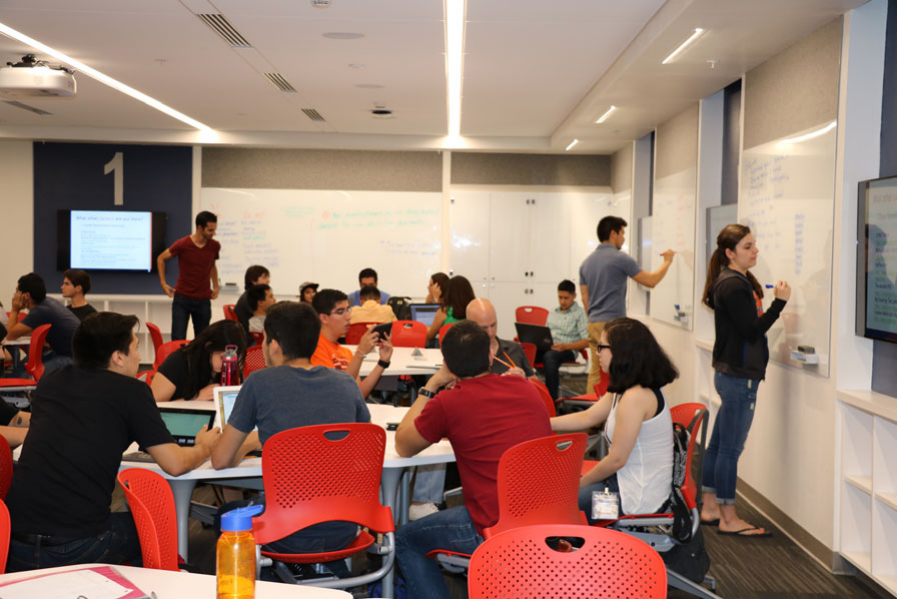
Active learning space created at UTEP through BUILDing SCHOLARS (The authors have received consent to publish from those in the photo)

Successful implementation of the student and faculty development innovations (described below) is facilitated by the creation of new learning spaces as well as the development and delivery of novel curricula (by faculty and postdoctoral research fellows) to support effective course-based undergraduate research experiences. BUILDing SCHOLARS created an active learning space designed based on the SCALE-UP model [6]. To create the space, we transformed 2000 square feet of building shell space into a 75-seat learning environment, centered on nine-student modular tables that readily separate into three smaller tables (Fig. 3). Each nine-student table has an independent whiteboard space, a video monitor and feed, a microphone, a high capacity cable internet connection (in addition to general Wi-Fi connectivity in the room), and power outlets to support both class and student electrical accessories. The instructor has full control of video input and display for each table, allowing her/him to share individual screens in any pattern. Students may connect their own devices into the network, or they may borrow BUILDing SCHOLARS tablets for class activities. The room also has teleconferencing capability for bringing in outside speakers (for various course-based and professional development activities) and/or remotely sharing activities with other classes or online students. BUILDing SCHOLARS uses the space for research foundations course (RFC) sections, non-laboratory-based research driven course (RDC) sections, and professional development workshops, among other uses. Faculty who have undergone training in both pedagogy and technology may also use the learning space for other classes, as scheduling of the RFCs permits. Evaluation data from student users indicate that the SCALE-UP space is highly effective relative to traditional learning environments because it facilitates collaboration and communication; makes learning more engaging, interactive and enjoyable; and fosters more physical and social comfort.

We are also enhancing research training infrastructure by investing in new laboratory spaces and postdoctoral fellows to support research-driven courses (RDCs). The new RDCs are designed by UTEP faculty (with BUILD postdoctoral fellows) based on their active research agendas in order to catalyze undergraduate participation in faculty members’ ongoing investigations. To initiate development of the RDCs, we identified interested, established faculty members at UTEP who were active health researchers, and worked with them to develop concepts for courses. We then hired postdoctoral fellows with demonstrated research experience in the course topic areas as well as interest in building their pedagogical expertise using discipline-based education research [7]. Together, faculty-postdoctoral fellow teams developed their RDCs, with each postdoctoral fellow assuming leadership in the delivery of the course to students. To ensure that all RDCs integrate authentic research experiences, each course syllabus undergoes pre-implementation review and each course is formally evaluated every term of delivery based on its particular research learning outcomes. These investments are enabling us to scale-up the impacts of undergraduate research training at UTEP, as the new curriculum is broadly benefitting UTEP students, not just BUILDing SCHOLARS trainees. In the academic year 2015–2016, the RFCs served 221 students and the RDCs served 220 students at UTEP; in 2016–2017, the RFCs and RDCs served 289 and 252 students, respectively, at UTEP.

We also recognize that in order to enhance the research training environment at UTEP over the long term, the research conducted by faculty in the biomedical sciences must improve such that (inter)national recognition and the continual acquisition of external funding become the norm. To contribute to that goal, BUILDing SCHOLARS has invested in key instruments in order to enhance the research capacities of large numbers of faculty mentors. Thus, in addition to major infrastructural investments in research teaching spaces, we have supported the acquisition of two instruments—a circular dichroism spectropolarimeter and a DNA sequencer—needed to advance the research programs of multiple faculty members, including current and prospective mentors to BUILDing SCHOLARS trainees. One instrument did not previously exist at UTEP, while the other was needed because existing instruments were being used at maximum capacity. Protocols to enable maintenance and maximum utilization of these instruments have been implemented.

### Institutionalizing and sustaining highly effective program elements

While BUILDing SCHOLARS activities are being supported initially by external funding, we designed them with prospects for eventual migration to other support. Ideally all of the successful interventions would be maintained through a dedicated endowment or specific donor, foundation, or corporate gifts, and we are working on developing these assets. At the same time, we recognize that some of our activities may need to be supported by more traditional grants and/or institutional operating budgets, so we are also developing strategies and plans to achieve sustainability for those activities.

Conceptually, we separate program funding needs into two categories: One-time expenditures vs. continuing operational costs. One-time expenditures include, for example, remodeling/equipping facilities or compensating faculty to develop new research training activities, based on emerging evidence that these interventions generate positive student developmental outcomes. Such expenditures are typically connected to hypothesis testing and new goals, and are therefore suitable for grants or other one-time funding sources. In contrast, continual operational costs are usually addressed as regular institutional budget items. For these latter expenses, our focus in terms of justifying institutionalization is not the total cost of the operations, but rather the cost relative to operations that are being replaced, as well as the added student developmental value. In the case of BUILDing SCHOLARS, some operations, such as the efficient use of the SCALE-UP facilities for larger classrooms, may result in net savings while enhancing student learning. Other operational costs, such as supplies for some RDCs, may far exceed those of the standard laboratory sessions that are replaced. In terms of prospects for institutionalization of such high cost program elements, we recognize that any increase in operational cost must be empirically linked to increased measures of student success, such as retention, graduation, and learning, outcomes that the University of Texas System links to each individual institution’s budget and supplemental allocations. Our evaluation efforts are designed to support our abilities to make such determinations and make the case for budget increase requests.

## Faculty development

The faculty development aims of BUILDing SCHOLARS are to: (1) coordinate faculty-to- faculty mentoring programs; and (2) create the institutional support structure to increase faculty research productivity and their ability to engage and prepare students for successful biomedical research careers. The ultimate goal is to strengthen the culture of research and mutually benefit undergraduate research training.

### Faculty-faculty mentoring programs

BUILDing SCHOLARS offers two faculty-to-faculty mentoring programs: (1) the supermentor program, which operates during the academic year, and (2) the summer sabbatical program, which runs during the summer. The goals of both programs are to enhance UTEP and pipeline partner faculty research skills, catalyze collaborative projects with research partner faculty, and increase competitiveness for external funding.

In the academic year program, faculty and postdoctoral fellows at UTEP and faculty from our pipeline partners apply to be mentored by a “supermentor,” who is an accomplished scholar from one of the research partner institutions. Supermentors are interested faculty members who are experts in acquiring external funding, publishing, and student mentoring. They are recruited by PIs at each research partner institution and listed in a directory published on the BUILDing SCHOLARS website. Supermentors receive an honorarium to incentivize their work. Interactions take place virtually and mentors spend up to five hours/month assisting their mentees, as per the contract that both parties initially sign. Five teams are funded each grant year. Faculty of all ranks have taken part in this program either as mentor or as mentee (with postdoctoral fellows participating as mentees only). These teams become competitive for other BUILDing SCHOLARS mechanisms, such as pilot grant, seed funding projects, and summer sabbaticals.

During the summer, we offer UTEP and pipeline partner faculty the opportunity for intensive face-to-face engagement with a faculty mentor/host at one of our research partner institutions. Since state institutions in Texas do not offer paid sabbaticals to their faculty, this is an excellent mechanism for UTEP faculty to develop their research skills or enter new research areas. Summer hosts are recruited by PIs at each research partner and listed in an online directory; a letter of support from a potential faculty host is required as part of the mentee’s application. Faculty mentees receive funds to cover travel, housing and living expenses during the 10 weeks of their sabbatical, and their hosts receive an honorarium to incentivize engagement.

### Institutional support for faculty

To enhance the development of faculty members as researchers, teachers and mentors, we offer compensation for research-driven course (RDC) development, pilot research funding, seed funding for teaching and mentoring activities, and opportunities to attend seminars and workshops by experts on undergraduate research, teaching and mentoring. We also offer support for postdoctoral fellows, funded by BUILDing SCHOLARS, to deliver the research-intensive curriculum.

BUILDing SCHOLARS is currently supporting the implementation of a total of 12 RDCs, which as indicated above are developed by faculty and postdoctoral fellows. All faculty who lead RDCs are provided one course release (or the equivalent in summer salary) during the development stage, a budget for equipment and supplies, as well as a postdoctoral fellow who co-develops and instructs the course.

Research pilot grants are competitively awarded based on an application process to support research teams of two or more individuals from different institutions to conduct innovative biomedical research. These awards aim to foment the development of ideas for transformative research projects that include undergraduate participation and to develop collaborative relationships among participating partner institutions, which lead to NIH proposal submissions. Ideally, BUILDing SCHOLARS trainees interface with these awards at UTEP or during the summer at one of the research partners.

Seed funding awards are competitively awarded to faculty for the purposes of expanding and scaling-up innovative mentoring and research curricular development activities, which are pillars of BUILDing SCHOLARS. UTEP or pipeline partner PIs, motivated to increase research opportunities for undergraduates, are eligible to receive seed funding awards. Seed funding projects focus on the creation of new research-intensive courses, mentoring activities and programmatic offerings, which enhance faculty and institutional capacities to support undergraduate research training experiences.

BUILDing SCHOLARS also hosts speakers and workshops focused on mentoring and engaging undergraduates in research to generate excitement and positively shape the mentoring culture at UTEP and beyond. Based on work published as part of the BUILDing SCHOLARS planning grant, the quality of mentoring was shown to play a critical role in UTEP student gains via undergraduate research experiences [8]. These workshops target faculty across the UTEP campus as well as those at pipeline and research partner institutions.

To support our postdoctoral fellows, we have instituted a training/development program that includes meeting biweekly to focus on professional development needs, drafting and continually refining individual development plans, funding travel for research presentations at conferences and professional development activities, and encouraging participation in relevant external training opportunities.

## Preparing students for biomedical research careers

The student development aims of BUILDing SCHOLARS are to: (1) recruit freshmen, sophomores, and juniors from UTEP as well as transfer students from pipeline partners to apply for student trainee scholarships; (2) retain scholarship students through graduation; (3) engage students in rigorous research and professional training that starts as early as the freshman level; (4) operate an intensive summer research program (SRP) for undergraduate students and faculty across partner institutions; and (5) coordinate peer mentoring and student-faculty research mentoring programs.

### Theoretical model orienting student development: Augmenting asset bundles

Our student development activities are founded upon a conceptual model for nurturing students from SURGes in their areas of greatest need in order to enable success in research careers. This “asset bundles” framework focuses on enhancing the scientific, social, and financial capital of our students. Johnson and Bozeman [9] identified five bundles of assets that should be developed through research training programs: (1) educational endowments, (2) science socialization, (3) network development, (4) family expectations, and (5) material resources. The training sequence and required extra-curricular activities in BUILDing SCHOLARS are designed to increase capacity in each of the bundles. (1) Educational endowments reflect the knowledge, skills, and abilities that students possess prior to participating in the program and are augmented through the summer boot camp, the summer research foundations course (RFC), the research driven courses (RDCs), and seminars and workshops that students are required to attend. (2) Science socialization is fostered through ethics training, common seminars and workshops, the common research-driven curriculum, and continued research experiences at UTEP and research partner institutions. A senior honors thesis in the format of a publishable journal article develops assets in terms of both educational endowments and science socialization. (3) Network development is nurtured through a “communities of practice” approach that strives to integrate mentoring across the network [10, 11]. Within their community of practice, students simultaneously have relationships with multiple members, which leads to their being identified as a novice within one relational sphere and a possible expert or mentor in another [10]. Acquiring expertise and recognition as “experts” helps BUILDing SCHOLARS trainees develop confidence and motivation, two key attributes for persistence in science [12]. (4) Family expectations are addressed through family participation with trainees at several key events, including the programmatic overview and BUILDing SCHOLARS contract presentation (prior to scholarship acceptance), the new trainee celebration and orientation, and the summer research program orientation. (5) Monetary resources are provided to the students in the forms of full tuition scholarships, monthly living stipends, paid summer research program experiences at research partner institutions and travel awards, which allows them to prioritize their own development as budding scientists rather than preoccupy themselves with personal finances.

### Student recruitment and retention

The BUILDing SCHOLARS scholarship is an annual award that includes full tuition for 15–18 credits per semester and a living stipend amount determined by the Ruth L. Kirschstein-NRSA Award Stipends [13]. The scholarship is available for up to four years. BUILDing SCHOLARS scholarship recipients are all enrolled at UTEP and denoted as “trainees”.

Students may become trainees in one of three ways: (1) as incoming freshmen recruited from high schools, (2) as rising sophomores and juniors already enrolled at UTEP, and (3) as rising sophomores and juniors transferring from our two-year pipeline partners. Table 3 shows planned UTEP trainee numbers for the four cohorts recruited for participation in academic years 2015–2016 through 2018–2019. Note the numbers depicted do not include students enrolled at pipeline partner institutions who participate in our SRP or those at EPCC. Such students are not recipients of the BUILDing SCHOLARS scholarship when participating in the SRP, although they can apply for it.

**Table 3:** BUILDing SCHOLARS scholarship students per year

																2019–2020			
												2018–2019					Cont.	New	Total
								2017–2018					Cont.	New	Total	FR	0	25	25
				2016–2017					Cont.	New	Total	FR	0	25	25	SOPH	25	5	30
2015–2016					Cont.	New	Total	FR	0	25	25	SOPH	25	5	30	JR	30	15	40
	Cont.	New	Total	FR	0	25	25	SOPH	25	5	30	JR	30	10	40	SR	40	0	40
FR	0	25	25	SOPH	25	5	30	JR	30	10	40	SR	40	0	40	Total	95	40	135
SOPH	0	10	10	JR	10	10	20	SR	20	0	20	Total	95	40	135				
JR	0	25	25	SR	25	0	25	Total	75	40	115								
SR	0	0	0	Total	60	40	100												
Total	0	60	60																

Notes: 2019–2020 is shown to illustrate maximum student participation is attained in 2018–2019; Cont. = continuing

In order to retain our trainees, we provide an integrated social and academic support system [14]. All trainees meet with the BUILDing SCHOLARS advisor once per semester or more frequently as needed. Those undergoing peer-mentor training also meet with each other and the peer-mentor training instructor on a weekly basis (see the “Peer-Mentoring Programs” subsection below for details). All students complete a weekly report online, in which they specify the number of hours spent on BUILD-related activities, as well as report good news and any concerns that emerged during the week. In this way, we are able to promptly respond to students’ issues. BUILDing SCHOLARS requires a 3.3 GPA to remain in good standing. When a trainee drops below 3.3, he/she has a planning meeting with program directors and staff where an individual recovery plan is developed. If the trainee follows the plan and is still below 3.3 after the next semester, he/she is granted a second semester of probation. Recovery plans may include required tutoring, counselling, and writing support. BUILDing SCHOLARS employs a science writer and tutors who can assist the trainee at no cost. The recovery plan is revised each semester that the trainee is on probation. Trainees who do not meet the GPA requirement after being on probation for two semesters are dismissed from the program. Evidence indicates that our recovery interventions are highly effective in terms of retaining students: thus far, 15 students have been on probation and only one was not retained after implementation of a two semester recovery plan.

### Research and professional development training

**Fig. 4:**
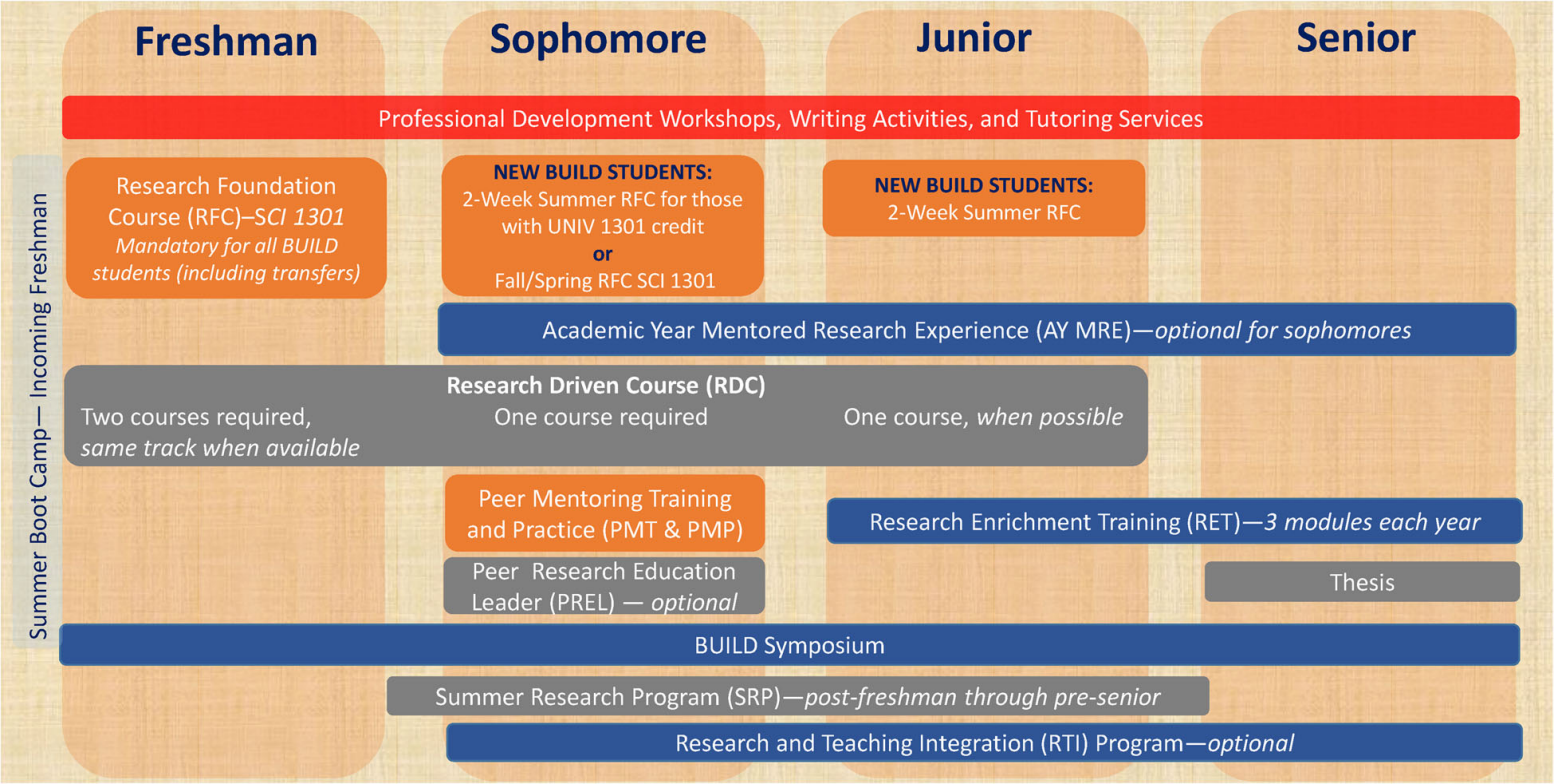
Overview of the BUILDing SCHOLARS student training sequence

The BUILDing SCHOLARS student training sequence, which is completed in addition to other degree plan requirements, is depicted in Fig. 4. It begins the summer before students begin their first semester as trainees. Entering freshmen participate in a three-week educational “boot camp” the summer before their first semester. The boot camp consists of preparation for calculus, statistics, writing, verbal communication, reading comprehension, and financial literacy (as recommended by the asset bundles approach [9]) using on-line modules [15] and face-to-face instruction. Necessary life skills for the college student as well as four hours of ethics training about proper inclusion of humans and animals in research, delivered by representatives from the Institutional Review Board (IRB) and Institutional Animal Care and Use Committee (IACUC), are also covered. Evaluation data (based on pre/post-testing) for both the 2015 and 2016 editions of the summer boot camp reveal highly successful outcomes in terms of changes in pre-test to post-test performance among students in calculus, statistics and writing.

During their first fall semester, trainees mandatorily enroll in a section of our RFC (or research foundation course sections of “Inquiry in Math & Science” (SCI 1301)), a core curriculum course at UTEP. Traditional sections of this course are designed to engage students in critical inquiry into one or more academic topics and introduce them to campus resources. The flexible curriculum allows the research foundations-specific sections, which are open to all students on campus, to focus on the fundamentals of the research process. All sections of the course are taught in the SCALE-UP space and implement a common curriculum designed to promote inquiry-based, student-centered learning. Participants in these courses use core concepts from engineering and the natural and socio-behavioral sciences to make interdisciplinary conceptual links. Students analyze primary literature and make connections among research findings and biomedical science and engineering advancements. In the process, they learn about the schematics and logical flow of journal articles, and the value of proper written and oral communication in establishing the trust upon which the entire research enterprise is founded. Finally, to hone their research skills, student teams propose basic research questions, design simple procedures and methodological protocols to answer them, and conduct final presentations of their work. In discussing real ethics cases in small groups, students learn about the responsible conduct of research and the protection of intellectual property. New entering sophomore and junior trainees who have already completed SCI 1301 or its equivalent, take part in a two-week compressed version of this course the summer before their first semester as trainees. Evaluation data for the RFCs based on student self-assessments (using a pre/post design) indicate that these courses significantly enhance science self-efficacy among students. For example, students experienced significant increases in self-rated knowledge regarding research-related topics/concepts as well as confidence in conducting research-related activities.

Trainees also enroll in two research-driven courses (RDCs) that offer students authentic research experiences and fulfill existing degree plan requirements. Most RDCs are at the freshman level and a few are offered at the junior/senior level. While meeting the learning objectives of existing traditional lab or lecture courses, RDCs immerse students in cutting-edge research projects that provide opportunities to make original disciplinary contributions while developing relevant skills, acquiring practical experience, and entering a community of practitioners. The aim of the RDCs is to help students form their identities as biomedical research scientists or engineers at an early stage of their academic development. Table 4 provides information on the BUILDing SCHOLARS RDCs, with topics relevant to each of the seven research nodes. These courses are also open to other undergraduates who want to participate, not just trainees. Through funding from the Howard Hughes Medical Institute and the National Science Foundation, other RDCs have been or are being implemented, and the two-to-three semester sequence (the RFC plus RDCs) are now coordinated through a new program called the Freshman Year Research Intensive Sequence (FYRIS). The intent is to institutionalize the program regardless of source of funding, given its early success with first-year student retention [12]. Evaluation data for the RDCs based on student self-assessments (using a pre/post design) indicate that all of these courses significantly increase students’ discipline-specific knowledge and confidence in conducting research.

**Table 4:** BUILDing SCHOLARS research-driven courses (RDCs)

Area	Course Type	Level	RDC Title
Biology	Lab	Fr I & II	Stop that Bacteria!
Chemistry	Lab	Fr I & II	Circadian Rhythm Genes and Proteins
Engineering	Lab	Fr I	mHealth Technologies
Engineering	Lab	Jr & Sr	Advanced mHealth Technologies
Psychology	Lecture	Fr I	Cultural Effects on Health Decisions
Psychology	Lecture	Soph	Behavioral Interventions in Medical Settings to Address Substance Abuse among Ethnic Minorities
Biology	Lab	Fr I & II	Antagonizing G-protein Coupled Receptors
Biology	Lab	Fr I & II	Characterization of Cellular Proteins Implicated in HIV-1 Replication
Chemistry	Lab	Jr & Sr	Proteins Folding Proteins: Understanding How Chaperonins Function
Sociology	Lecture	Fr I	Interpersonal and Institutional Factors affecting Reproductive Health Practices on the US-Mexico Border
Engineering	Lab	Jr & Sr	Biomaterial Implantology
Engineering	Lab	Jr	MEMS-based Microfluidic Devices for Biomedical Applications
Psychology	Lecture	Fr I	Psychobiology of Addiction Behavior
Social Work	Lecture	Jr & Sr	Tackling the Root Causes of Heath Disparities

Once students complete the RFC/RDC sequence, and/or begin participating in mentored research activities, they are required to attend a series of themed professional development (PD) workshops, including (but not limited to) “Responsible Conduct of Research,” “Preparing Abstracts,” “Introduction to NIH and NIH Resources for Students,” “Entrepreneurship,” and “Developing Your Professional Path.” Additionally, statistics-focused workshops open to the campus community are provided. The sequence of PD workshops is designed to match the level of the student. The BUILDing SCHOLARS training culminates in the development of a senior honors thesis. As juniors and seniors, trainees work directly with a mentor on developing a project for their honors thesis. This document, which is prepared by the trainee in the format of a journal article and reviewed by two faculty members, is expected to be of high enough quality to be submitted for peer-reviewed publication.

#### Summer & academic year mentored research programs

BUILDing SCHOLARS offers all student trainees summer research experiences while they are in the program. Each year, approximately two-thirds of trainees attend one of the 12 research partner institutions either to be integrated into their existing summer research programs or to participate in newly-created BUILDing SCHOLARS-specific programs. The other one-third of trainees stay at UTEP for summer research and are joined by pipeline partner students coming to UTEP for their first research experience. If they remain in good standing, these pipeline partner students are invited back for a second summer research experience. Before beginning their research placement, pipeline partner students attend a one-week version of the BUILDing SCHOLARS research foundations course (RFC). We implemented this pre-training approach in response to our capacities/needs assessment, through which it was requested by many prospective faculty mentors. The UTEP-based summer research program includes the “Entering Research” mentee training [16]. Wherever their placement, all students reconvene to deliver poster presentations about their summer research program projects to the PIs from all partner institutions at the annual Fall partnership meeting at UTEP.

BUILDing SCHOLARS coordinates mentor-mentee matching across all summer research institutions using a profile system created in Chronus©, a mentoring software platform that facilitates delivery of the multi-institutional program. Faculty mentors recruited at each institution are asked to complete a profile; students also complete profiles. We then employ an algorithm based on research interests, discipline, student preference for location, and the number of slots available in order to match mentors with mentees. Mentors and mentees in our summer program are connected through a virtual “Connection Plan” in Chronus©. Similar to online courses, Connection Plans enable us to coordinate and oversee a program involving myriad institutions. The platform was piloted for the 2016 SRP with very good matching results. Student placement numbers in the SRP through summer 2017 are provided in the “A Partnership Network” section above.

Mentors are required to complete the NRMN-affiliated online training course “Optimizing the Practice of Mentoring” [17]. We expect that this focus on enhancing the quality of mentored research experiences will prove effective, since mentoring quality has been shown to increase UTEP student gains through undergraduate research experiences [8].

During the academic year, BUILDing SCHOLARS operates a faculty-mentored research program for our UTEP trainees. This program parallels the summer program in terms of design. All trainees enroll as juniors and seniors, along with motivated self-selected sophomores. We use Chronus© to match the students with UTEP mentors and virtual Connection Plans to deliver the mentoring program, including prompts for the senior thesis. In 2015–2016, 11 BUILD trainees were placed in new academic year faculty-mentored research experiences, and during 2016–2017, 48 BUILD trainees have been placed in new academic year faculty-mentored research experiences.

#### Peer-mentoring programs

All freshmen and sophomore trainees are required to participate in the peer-mentoring program, in which sophomore mentors provide academic, social, and personal support to freshmen mentees. The peer-mentoring program nurtures sophomore mentors’ identities as leaders while also supporting the psychosocial needs of our freshmen. Sophomore trainees attend weekly mentoring classes (2 h/week) to improve their mentoring, leadership, and emotional intelligence skills. To match peer mentors with mentees, we use Chronus© and the matching algorithm is more focused on social aspects than research interests. Each peer mentor has one or two mentees with whom they meet at least twice per month. Peer mentors also have the opportunity to serve as “Peer Research Education Leaders,” whereby they assist in the RDCs and RFCs. This opportunity is offered to peer mentors who are selected by the RDC and RFC instructors.

## Site level evaluation design and early outcomes

BUILDing SCHOLARS employs an internal team to evaluate all of our activities. Formative and summative evaluations examine and assess project implementation, progress, and effectiveness toward meeting stated goals and objectives. The formative evaluations focus on project implementation and operation processes from the first to the final year of funding. The evaluation team attends team meetings and meets regularly with the program directors to document planned activities (i.e. student recruitment, courses, professional development workshops/seminars, etc.) and coordination efforts. Quantitative and qualitative data gathered from program participants, institutional records and information from the meetings are used to assess whether the program progresses as planned and whether any unexpected issues and/or challenges surface. A main goal of the formative evaluations is to identify barriers/challenges to meeting program objectives as early as possible in order to develop strategies to effectively improve the quality and continued progress of the program. Results from formative evaluations in particular are used by program directors to inform revisions to program components and elements to have more positive impacts on students and faculty, as well as on the institutional environments at UTEP and partner institutions.

The summative evaluation, on the other hand, focuses on the overall impact that the program has on student and faculty participants, as well as on the institution in general. Special attention is given to identifying and monitoring agreed upon Hallmarks of Success (see [18], this volume) that are relevant to the development and progress of participating students and faculty; program effects on specific institutional characteristics are also examined. While a main program evaluation limitation is the inability to use randomly controlled trials in order to infer intervention cause and effect, in specific instances, careful selection and statistical control of factors that may also influence change in the Hallmarks of Success provide information about the association between program participation and expected outcomes. Together, the formative and summative evaluations serve to determine the overall efficacy of the BUILDing SCHOLARS program in meeting its goals and objectives. Finally, our internal evaluation team has established a collaborative relationship with the NIH-funded Coordination and Evaluation Center (CEC) for tracking and evaluating BUILDing SCHOLARS site-specific activities, and for evaluating the efficacy of interventions across the 10 BUILD sites in the Diversity Program Consortium.

To summarize early outcomes, evaluation data indicate that the program elements described above have been highly successful across the partnership in terms of: scaling up the availability of top-flight biomedical research experiences for undergraduate students; delivering a new research-based curriculum that promotes student development while increasing undergraduate access to authentic research opportunities that, in turn, feed into faculty research; and promoting institutional development to support students, postdoctoral personnel and faculty members through infrastructure improvements, professional development programming, and funded research and mentoring opportunities. Because undergraduate trainees comprise the focal group of BUILDing SCHOLARS, it is important to note that the program has been highly successful in terms of retention. At the time of writing, BUILDing SCHOLARS has funded 92 UTEP trainees via scholarships. Of those, 87 have been retained or have graduated, which implies that they have excelled in program activities and coursework (by maintaining a GPA of 3.3 or higher); only five trainees have left the program, all of whom have remained enrolled at UTEP. The high rate of retention among trainees attests to both their exceptional quality and the strong support structure instituted by BUILDing SCHOLARS.

## Unique features, challenges, and potential contributions

A cornerstone of BUILDing SCHOLARS is the early intervention approach, which addresses the attrition problem facing UTEP and the nation. Since many students exit STEM training pathways during their freshman and sophomore years, research experiences within those years are critically important to bolster persistence [12], as students become practitioners early-on and understand what conducting authentic scientific research is like. UTEP has a strong tradition of undergraduate research engagement, which contributes significantly to successful student outcomes [19]. However, as is the case at the vast majority of U.S. universities, prior to BUILDing SCHOLARS, undergraduate research opportunities at UTEP were almost exclusively offered to upper division students. At UTEP and elsewhere, factors such as high cost and space demands for research training constrain the degree to which faculty-mentored undergraduate research can be scaled-up to improve retention in the early undergraduate years (and even in later years), especially in the traditional model of conducting research in a faculty mentor’s lab. Course-based Undergraduate Research Experiences (CUREs) early in students’ undergraduate careers have shown to enhance retention and persistence and resolve some of the challenges inherent in the traditional undergraduate research model [12]. Through the integration of a new research-driven curriculum that primarily targets early undergraduate course requirements, BUILDing SCHOLARS is stimulating student motivation, skill-development, and confidence, with the goal of increasing retention, performance, and, ultimately, persistence in terms of progression toward successful careers. In addition to addressing attrition, early interventions like our RDCs, which are open to all students on campus, help to steer more students at an earlier stage toward biomedical research careers, and thus, increase the number of talented students in the pipeline. The development of other RDCs for junior and senior students targets the space limitations that prohibit some interested students from being able to receive research training in faculty members’ research labs and also helps recruit students overlooked in the early stages but who persisted in their majors.

The fundamental challenge with transporting an early intervention model to other institutional contexts, however, is that it necessitates major investments in new curriculum and infrastructure. Such institutional transformations challenge the traditional course offerings and are currently cost-prohibitive at UTEP and most U.S. universities. For example, as UTEP undertakes its laudable “access and excellence” mission, which emphasizes open acceptance and low tuition and fees, external funding is a must. Thus, federal and private funders have to be called upon to provide resources. The long-term goal would be to establish the curricular, physical, human and programmatic infrastructures across an array of suitable universities to first scale-up and then sustain the impacts of undergraduate research training for students from underrepresented groups. At a minimum, this would demand the development of new research-based courses by faculty members, the creation of new pedagogical spaces to implement those courses, the provision of materials and supplies to sustain those courses (which are usually more expensive than those required for traditional course offerings), and the hiring and training of skilled staff needed to support the curricular innovations.

Second, BUILDing SCHOLARS emphasizes continuous research experiences and academic enrichment for students, in order to promote persistence and enhance development. Our student development model targets the critical areas in which students from SURGes need additional support to enhance their achievement in research careers [9]. It is a long-term, multi-dimensional commitment to student training that contrasts with the prevailing short programmatic (e.g., summer or capstone) approach to undergraduate research engagement, which generates positive albeit limited student developmental benefits. Academic enrichment starts for many BUILDing SCHOLARS trainees before they begin their first course. Upon graduating, trainees who began as freshmen or sophomores gain over 2000 h of research experience outside of their research-intensive coursework. They present original research to the academic community and many will publish results in peer-reviewed outlets. Thus, trainees are being prepared to gain admission to highly competitive graduate programs, and excel in their doctoral studies and beyond.

Third, BUILDing SCHOLARS adopts a scalable approach and a focus on providing access for transfer students in order to broaden impacts and ensure sustainability. We are not simply serving 180 scholarship students at UTEP in isolation. The research foundations courses (RFCs) and RDCs are open to all UTEP students and are being institutionalized; similar courses have been launched at pipeline partner institutions. We are also committed to transitioning transfer students into BUILDing SCHOLARS upon their arrival at UTEP from two-year institutions. Two-year colleges enroll over half of all U.S. undergraduates and most students from groups traditionally underrepresented in STEM fields start at two-year colleges. Transfer students comprise nearly 40% of UTEP undergraduate enrollees and more than half of our graduating seniors; EPCC is the institution of origin for nearly three-quarters of UTEP’s transfer students. Students from EPCC and other pipeline partners are being provided transitional pathways (by way of reserved scholarships) into BUILDing SCHOLARS. The focus on scalability and facilitating transitions from pipeline partners serves to expand research opportunities for emerging biomedical scientists across the U.S. Southwest and broaden the pool of talented students from diverse backgrounds.

Fourth, unlike many current undergraduate research training programs at UTEP and elsewhere [20, 21], BUILDing SCHOLARS fully integrates social and behavioral science. This addresses an important need in biomedical science training for the next generation, since social disparities are becoming increasingly central to health scholarship [22]. NIH research funding priorities have increasingly emphasized social and behavioral science projects, but training programs in those areas have lagged behind [23]. BUILDing SCHOLARS also fully integrates biomedical engineering, and it is one of a few BUILD sites to do so. Our disciplinary inclusiveness casts a wide net for attracting students from 24 majors, including those who would otherwise slip through the cracks, as well as faculty from diverse disciplinary backgrounds with varied expertise.

Fifth, BUILDing SCHOLARS harnesses that inclusiveness by promoting an explicitly transdisciplinary framework—based on topical nodes rather than disciplines—in order to stimulate cross-disciplinary interactions and innovative collaborations.

Sixth, BUILDing SCHOLARS emphasizes providing faculty (especially those at an early career stage) with training in research pedagogy and mentoring, in order to improve their ability to engage and prepare students, as well as an array of resources to enhance their research productivity. The focus on faculty development distinguishes BUILDing SCHOLARS from the vast majority of student training programs. At UTEP, there are over 509 tenure-track/tenured faculty members (as of spring 2016). Faculty members are primarily non-Hispanic white (52%), but 27% are Latina/o (Hispanic); 15% are non-Hispanic Asian, 2% are non-Hispanic Black, and 35% are female [24]. A serious challenge to scaling-up and sustaining the impacts of undergraduate research training is the need to recruit and retain highly interested and capable faculty to mentor students. As part of the BUILDing SCHOLARS planning grant, we analyzed factors influencing faculty motivation to mentor undergraduates in research, which is enabling us to more effectively promote faculty participation. This includes pre-training students prior to their mentored-research experiences, providing opportunities for extracurricular faculty-undergraduate student interactions, and increasing faculty awareness of the positive impacts of mentoring underrepresented minority students [25, 26]. We also recognize that the development of junior faculty from underrepresented backgrounds has the potential to create a ‘halo effect,’ and may prove the most expedient avenue for achieving the transformative goals of the NIH BUILD initiative. Thus, targeting junior faculty with resources for enhanced research engagement and student training is one vehicle through which BUILDing SCHOLARS is striving to increase the diversity of the NIH-funded workforce.

In sum, BUILDing SCHOLARS has unique and innovative features designed to increase the pool of well-trained students in biomedical fields from underrepresented groups and communities who pursue graduate studies, research careers, and successfully compete for NIH funding. The greatest challenges we face derive from the ambitiousness of the program, but inclusive science requires high aims and stretching beyond current accomplishments to develop biomedical talent for SURGes.
